# Anti-inflammatory effects of *Zea mays* L*.* husk extracts

**DOI:** 10.1186/s12906-016-1284-9

**Published:** 2016-08-19

**Authors:** Kyung-Baeg Roh, Hyoyoung Kim, Seungwoo Shin, Young-Soo Kim, Jung-A Lee, Mi Ok Kim, Eunsun Jung, Jongsung Lee, Deokhoon Park

**Affiliations:** 1 Biospectrum Life Science Institute, Eines Platz 11th FL, 442-13 Sangdaewon Dong, Seoungnam City, 462-807 Gyeonggi Do Republic of Korea; 2 Department of Genetic Engineering, Sungkyunkwan University, 2066 Seobu Ro, Suwon City, 164-19 Gyeonggi Do Republic of Korea

**Keywords:** *Zea mays* L, iNOS, COX-2, NF-kB, ICAM-1, AP-1

## Abstract

**Background:**

*Zea mays* L. (*Z. mays*) has been used for human consumption in the various forms of meal, cooking oil, thickener in sauces and puddings, sweetener in processed food and beverage products, bio-disel. However, especially, in case of husk extract of *Z. mays*, little is known about its anti-inflammatory effects. Therefore, in this study, the anti-inflammatory effects of *Z. mays* husk extract (ZMHE) and its mechanisms of action were investigated.

**Methods:**

The husks of *Z. Mays* were harvested in kangwondo, Korea. To assess the anti-inflammatory activities of ZMHE, we examined effects of ZMHE on nitric oxide (NO) production, and release of soluble intercellular adhesion molecule-1 (sICAM-1) and eotaxin-1. The expression level of inducible nitric oxide synthase (iNOS) gene was also determined by Western blot and luciferase reporter assays. To determine its mechanisms of action, a luciferase reporter assay for nuclear factor kappa B (NF-kB) and activator protein-1 (AP-1) was introduced.

**Results:**

ZMHE inhibited lipopolysaccharide (LPS)-induced production of NO in RAW264.7 cells. In addition, expression of iNOS gene was reduced, as confirmed by Western blot and luciferase reporter assays. Effects of ZMHE on the AP-1 and NF-kB promoters were examined to elucidate the mechanism of its anti-inflammatory activity. Activation of AP-1 and NF-kB promoters induced by LPS was significantly reduced by ZMHE treatment. In addition, LPS-induced production of sICAM-1 and IL-4-induced production of eotaxin-1 were all reduced by ZMHE.

**Conclusions:**

Our results indicate that ZMHE has anti-inflammatory effects by downregulating the expression of iNOS gene and its downregulation is mediated by inhibiting NF-kB and AP-1 signaling.

## Background

Inflammation is a biological reaction mediated by inflammatory cells in response to cellular injuries. Although various types of cells are involved in the inflammatory reaction, macrophages are well known to play a central role in regulating the production of pro-inflammatory mediators. Inducible nitric oxide synthase (iNOS), one of inflammatory mediators, has been involved in the regulation of inflammatory responses. iNOS is an inducible enzyme and mediates similar pathological processes [1]. The production of nitric oxide (NO) is mediated by three types of nitric oxide synthase (NOS) such as endothelial NOS (eNOS), inducible NOS (iNOS) and neural NOS (nNOS) [2]. Among them, iNOS is involved in both regulatory and detrimental processes [3]. During the inflammation response, overproduced NO may exert cytotoxic effects [4].

NF-kB, one of transcriptional mediators, plays a major role in regulating the inflammatory responses by upregulating the level of various inflammatory mediators [5]. The activation of NF-kB induces the expression of these pro-inflammatory genes, including various inflammatory cytokines and genes encoding cyclooxygenase-2 (COX-2) and iNOS [6, 7]. Another transcription mediator, AP-1 also upregulates transcription of inflammatory genes [8]. Mitogen-activated protein kinases (MAPKs) can activate transcription mediators such as NF-kB and AP-1, consequently inducing the expression of pro-inflammatory mediators of extracellular stimuli [9].

*Zea mays* L. (*Z. mays*), corn or maize which is a annual grass in the *Poaceae* (grass family) that originated in Central America, is one of the main three cereal crops grown in the world, along with rice (*Oryza sativa*) and wheat (*Triticum* spp.). Corn is used for human consumption such as meal, cooking oil, thickener in sauces and puddings, inexpensive sweetener in processed food and beverage products, bio-disel and so on. Despite its wide spread use, there have been no reports which demonstrate its biological activities. Recently, it has been reported that corn possesses antiadhesive activity against uropathogenic *E. coli* [10], as well as antioxidant and antimutagenic activities [11]. In addition, no studies have examined the effects of *Z. mays* on inflammation-associated gene expression.

Therefore, we investigated the inhibitory effects and mechanisms of *Z. mays* against inflammatory signals and demonstrated that *Z. mays* inhibits LPS-induced inflammatory reactions through inactivation of NF-kB and AP-1 pathway in RAW264.7 cells.

## Methods

### Preparation of *Z. mays* extract

*Z. Mays* was harvested in Gangwon-do, Korea, from June to August and authenticated by Dr. Yong-Hwan Jung, Jeju Biodiversity Research Institute, Jeju Techno Park, Korea, where a voucher specimen (Voucher No. JBRI 140924–01). The dried powders from the whole plant (150 g), flag leaf (150 g), husk (150 g), cob (150 g), kernel (150 g), silk (150 g), tassel (150 g), and stalk (150 g) of *Z. Mays* were extracted with 70 % ethanol for 24 h, and the extract was incrassated by a rotary evaporator for 3 h. To remove the ethanol from the extract, it was mixed with water and incrassated again. Subsequently, the extract was filtered using filter paper and frozen on a freezing tray for 48 h. Freeze-drying powder of whole plant (21.3 g), flag leaf (21.0 g), husk (31.2 g), cob (6.3 g), kernel (11.8 g), silk (27.9 g), tassel (7.9 g), and stalk (20.7 g) were dissolved in DMSO for the experiments.

### Cell culture and reagents

Mouse macrophage cell line, RAW264.7 was obtained from the Korean Cell Line Bank (KCLB, Seoul, Korea). The cells were maintained in RPMI 1640 (HyClone, Logan, UT, USA), containing 10 % fetal bovine serum (FBS, Gibco, Carlsbad, CA, USA) and 1 % penicillin/streptomycin (Invitrogen, Carlsbad, CA, USA), at 37 °C, under 5 % CO_2_. NIH/3 T3 mouse fibroblast cell line was maintained in DMEM (HyClone, Logan, UT, USA), containing 10 % FBS and 1 % penicillin/streptomycin at 37 °C, under 5 % CO_2_. Lipopolysaccharides (LPS) and Griess reagent were obtained from Sigma Aldrich (St. Louis, MO, USA). Mouse IL-4 was purchased from eBioscience (San Diego, CA, USA). Inducible nitric oxide synthase (iNOS) antibody was purchased from Millipore Corporation (Beverly, MA, USA).

### Cell viability assay

Cell viability was measured using the MTT (3-[4,5-dimethylthiazol-2-yl]-2,5-diphenyltetrazolium bromide; USB Corp., Cleveland, OH, USA) assay. Cells were plated in triplicate wells of 24-well plates, and cultured for 24 h. The cells were then treated with samples for 24 h, under a serum-free condition. Then, MTT reagent (1 mg/ml) was added to each well, and the cells were incubated for 3 h. The medium was removed, and the cells were solubilized with dimethyl sulfoxide (DMSO, Sigma, St. Louis, MO, USA). The absorbance was measured by spectrophotometer at a wavelength of 570 nm.

### Nitric oxide determination

The concentration of nitric oxide (NO) in the culture supernatants was determined as nitrite, a major stable product of NO. The cells were plated in triplicate wells of 24-well plates and incubated overnight. The cells were then treated with samples for 24 h. The cell culture supernatants were incubated with Griess reagent for 30 min. The absorbance was measured by a spectrometer at a wavelength of 540 nm and calculated against a sodium nitrite standard curve.

### Western blotting

iNOS protein levels were measured by western blotting. The protein extracts were loaded on a NuPAGE Novex 10 % Bis-Tris Gel (Invitrogen, Carlsbad, CA, USA) and transferred to a nitrocellulose membrane. The membranes were blocked with 5 % bovine serum albumin (BSA, Sigma, St. Louis, MO, USA) for 1 h and then incubated with primary antibodies, followed by incubation with horseradish peroxidase (HRP)-conjugated anti-mouse IgG secondary antibody and detected using chemiluminescent HRP substrate (SurModics, Eden Prairie, MN, USA).

### Transient transfection and luciferase assay

RAW264.7 cells were transfected with the iNOS, and NF-kB luciferase reporters using SuperFect® Transfection Reagent (Qiagen, Hilden, Germany). After 24 h of incubation, the cells were incubated in the presence or absence of *Z. mays* husk extract (ZMHE) induced by LPS for 24 h. The cells were then harvested and lysed, and the supernatants were assayed for their luciferase activity using a Dual Luciferase Assay System (Promega, Madison, WI, USA), and an Infinite® 200 PRO luminometer (Tecan, AG, Männedorf, Switzerland).

### Enzyme-linked immunosorbent assay (ELISA)

Eotaxin-1 concentrations were quantified in culture supernatants of NIH/3 T3 after treatment of ZMHE induced by IL-4 using a commercially available ELISA kit (eBioscience, USA). Cell culture supernatants were collected 24 h after treatment with ZMHE, and assayed for eotaxin-1. Soluble intercellular adhesion molecule-1 (sICAM-1) concentrations were quantified in culture supernatants of RAW264.7 after treatment of ZMHE induced by LPS using a commercially available ELISA kit (R&D systems, Inc., Minneapolis, MN, USA). Cell culture supernatants were collected 24 h after treatment with 50 ppm ZMHE, and assayed for sICAM-1. The standard curve was linearized and subjected to regression analysis. The eotaxin-1 and sICAM-1 concentrations were determined using a standard curve.

### Determination of total phenolic contents

The content of total phenols was determined by spectrophotometer, using gallic acid as standard, according to the method described by the International Organization for Standardization (ISO) 14502–1. Briefly, an aliquot of the diluted extracts (1.0 ml) was transferred into a separate tubes containing a 5.0 ml of a 1/10 dilution of Folin-Ciocalteu’s reagent in water. Then, a sodium carbonate solution (4.9 ml, 7.5 % w/v) was added. The tubes were then allowed to stand at room temperature for 60 min and then measured absorbance against water at a wavelength of 765 nm. Total phenolic contents was expressed as gallic acid equivalents in mg/g extract. The concentration of polyphenols in extracts was derived from a standard curve of gallic acid.

### Determination of total flavonoids

Samples (0.25 ml of the extracts) was added containing distilled water (1 ml) and then 5 % NaNO_2_ (0.075 ml), 10 % AlCl_3_ (0.075 ml), and 1 M NaOH (0.5 ml) were added sequentially at 0.5, and 6 min. Finally, the volume of the reacting solution was adjusted to 2.5 ml with double-distilled water. The absorbance of the solution was measured by spectrophotometers at a wavelength of 410 nm. Total flavonoids were expressed as quercetin equivalents in mg/g extract.

### Statistical analysis

All data are expressed as means ± standard deviations. Statistical significance of the data was determined using a Student’s *t*-test. A *P* < 0.05 was considered to be significant.

## Result

### *Z. mays* husk extract suppresses NO production in LPS-induced RAW264.7 cells

**Fig. 1:**
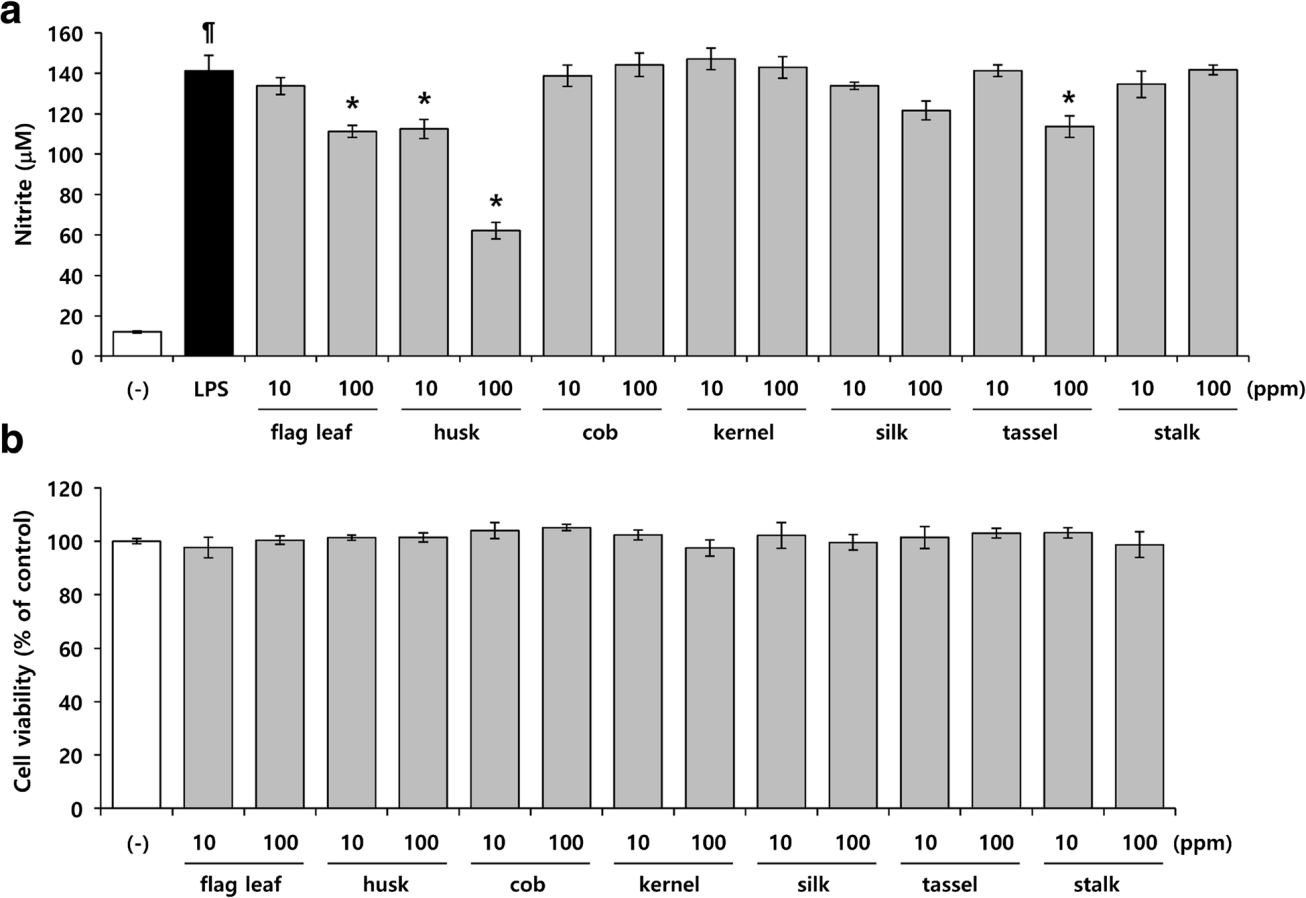
Effects of *Z. mays* extracts on NO production in LPS-induced RAW264.7 cells. **a** The cells were pretreated with the indicated concentrations of seven parts of *Z. mays* extract for 1 h and then further incubated with LPS (200 ng/ml) for 24 h. The amount of NO production was then determined using Griess assay. **b** Cell viability was measured by MTT assay. The results are mean ± standard deviation (SD) (*n* = 3). *P* < 0.01 vs. LPS-untreated control. *P < 0.01 vs. LPS-treated control

To determine anti-inflammatory effects of *Z. mays*, seven different aerial parts (husk, flag leaf, cob, kernel, silk, tassel, and stalk) of *Z. Mays* were analyzed for nitric oxide inhibition activity. Among the different aerial parts of the *Z. Mays*, the husk extract exhibited best nitric oxide inhibition activity (56 %) followed by leaf extract (21 %), tassel extract (19 %) and silk extract (13 %) respectively, compared to control (Fig. 1a). Cytotoxicity was not observed in seven parts of *Z. Mays* extracts at the concentration range of 10 ~ 100 ppm, when the cells were incubated for 24 h (Fig. 1b). We also examined the nutritional composition of ZMHE. As shown in Table 1, the content of carbohydrate was the highest (53.04 g/100 g). In addition, the total phenol and flavonoid contents of ZMHE was 5.92 ± 0.104 mg gallic acid equivalent (GAE) /100 g extract and 35.40 ± 1.41 mg quercetin equivalent (QUE) /100 g extract, respectively (Table 2).

**Table 1:** Nutrient composition of ZMHE

Nutrient composition		g/ 100 g
Fat		1.16
Protein		5.98
Ash		18.21
Fibre		0.07
Carbohydrate	Fructose	14.95
	Glucose	13.95
	Sucrose	24.14
	Lactose	0.00
	Maltose	0.00
	Total	53.04

**Table 2:** Total phenolic and flavonoid contents in ZMHE

Total phenolic contents (mg GAE/g)	Total flavonoid contents (mg QUE/g)
5.92 ± 0.104	35.40 ± 1.41

### ZMHE inhibits iNOS expression in LPS-induced RAW264.7 cells

**Fig. 2:**
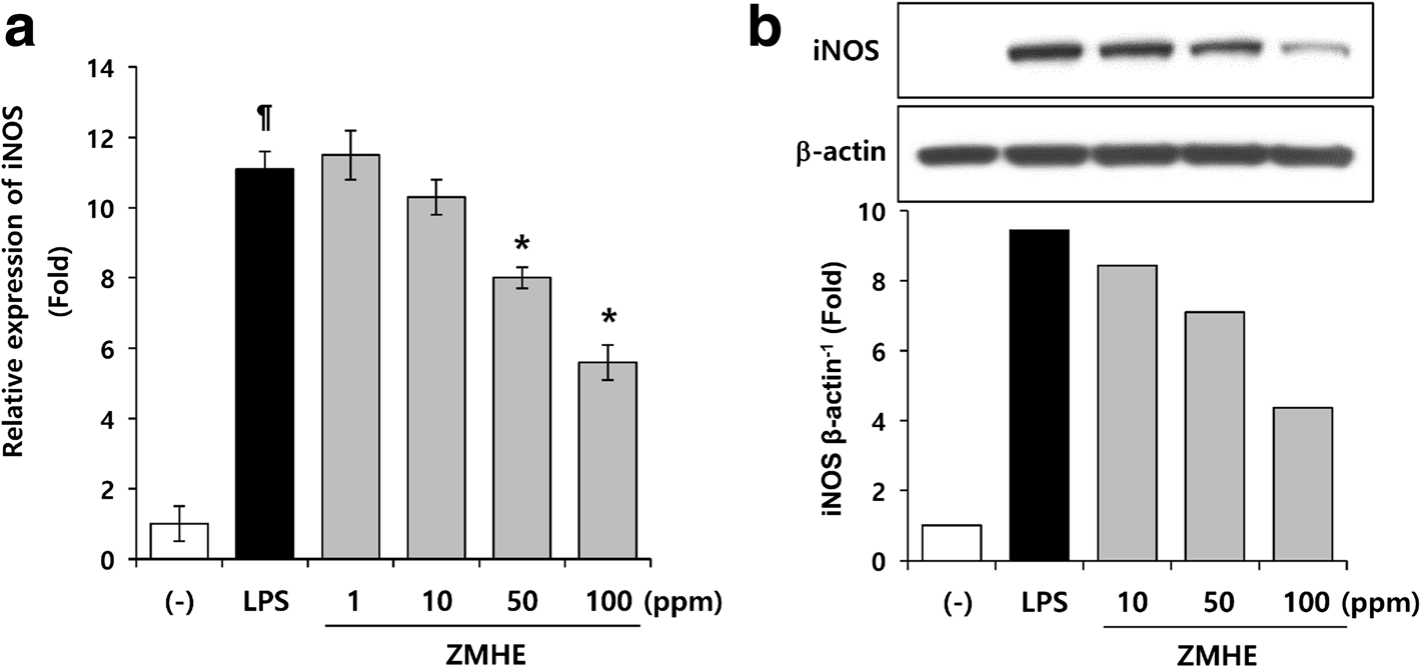
Effects of ZMHE on iNOS expression in LPS-induced RAW264.7 cells. **a** The iNOS luciferase reporter vector was transfected into RAW264.7 cells and cultured for 24 h. The cells were pretreated with ZMHE for 1 h and then stimulated with LPS (200 ng/ml). Luciferase activity was calculated against an LPS-unstimulated control. **b** The cells were pretreated with the indicated concentrations of ZMHE for 1 h and then further incubated with LPS (200 ng/ml). After 24 h incubation, the cell lysates were prepared and then subjected to Western blot analysis. The bands for iNOS were detected, and normalized to that of β-actin. Densitometric analysis was performed by using ImageJ program. The results are mean ± standard deviation (SD) (*n* = 3). *P* < 0.01 vs. LPS-untreated control. *P < 0.01 vs. LPS-treated control

iNOS which is primary responsible for the production of NO in inflammatory processes, is not typically expressed in resting cells but induced by certain cytokines or microbial products [2]. Therefore, downregulation of iNOS expression could be a chemotherapeutic method to improve inflammatory symptoms. Among seven different *Z. Mays* extracts, we selected ZMHE which showed the best NO inhibitory activity and then investigated its effect on LPS-induced iNOS expression in RAW264.7 cells. A luciferase reporter assay and Western blot were performed to measure iNOS expression. As shown in Fig. 2a, LPS-induced activation of iNOS promoter was significantly inhibited by ZMHE (5.6 ± 0.47) compared to LPS-treated group (11.1 ± 0.5). Consistent with this result, LPS-induced iNOS expression was also significantly inhibited by ZMHE at protein level (Fig. 2b). These results indicate that the ZMHE-mediated inhibition of NO production is associated with the suppression of iNOS expression at the transcriptional level.

### ZMHE effects are mediated by inhibiting AP-1 and NF-kB

**Fig. 3:**
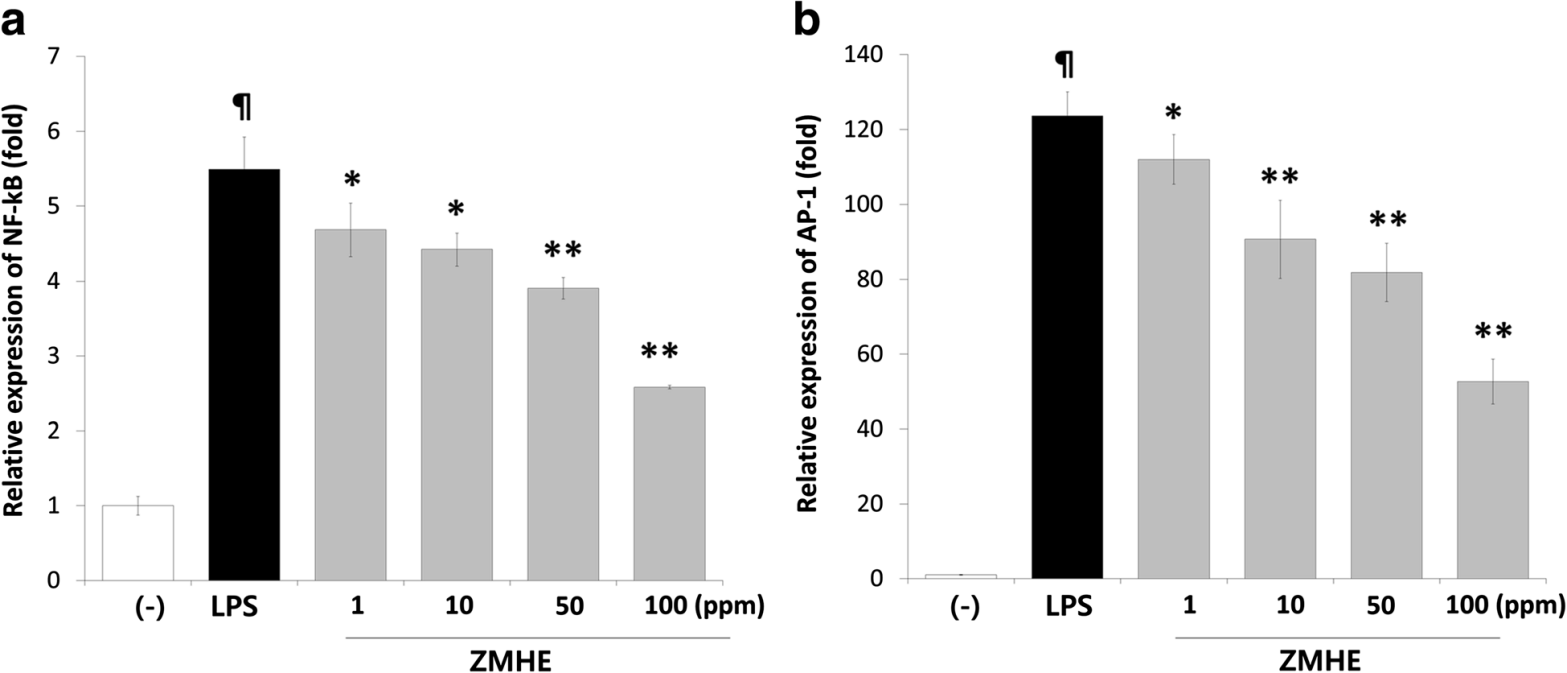
Effects of ZMHE on activation of NF-kB and AP-1 in LPS-induced RAW264.7 cells. The NF-kB (**a**) or AP-1 (**b**) luciferase reporter vectors was transfected into RAW264.7 cells and cultured for 24 h. The cells were pretreated with ZMHE for 1 h and then stimulated with LPS (200 ng/ml). Luciferase activity was calculated against an LPS-unstimulated control. The results are mean ± standard deviation (SD) (*n* = 3). *P* < 0.01 vs. LPS-untreated control. *P < 0.05 vs. LPS-treated control. **P < 0.01 vs. LPS-treated control

AP-1 and NF-kB regulate the expression of the target genes that are involved in inflammation [12], and plays an important role in the expression of iNOS [2] and eotaxin-1 [13]. Thus, we investigated the effects of ZMHE on activation of NF-kB and AP-1 using the luciferase reporter assay. In this study, ZMHE suppressed activation of NF-kB (2.58 ± 0.02) compared to LPS-treated group (5.49 ± 0.43). AP-1 promoter activity was also inhibited by ZMHE (52.67 ± 6) compared to LPS-treated group (123.62 ± 6.45) (Fig. 3a, b). These results suggest that the effect ZMHE effect is dependent on NF-kB and AP-1 signalings.

### ZMHE inhibits IL-4-induced eotaxin-1 expression

**Fig. 4:**
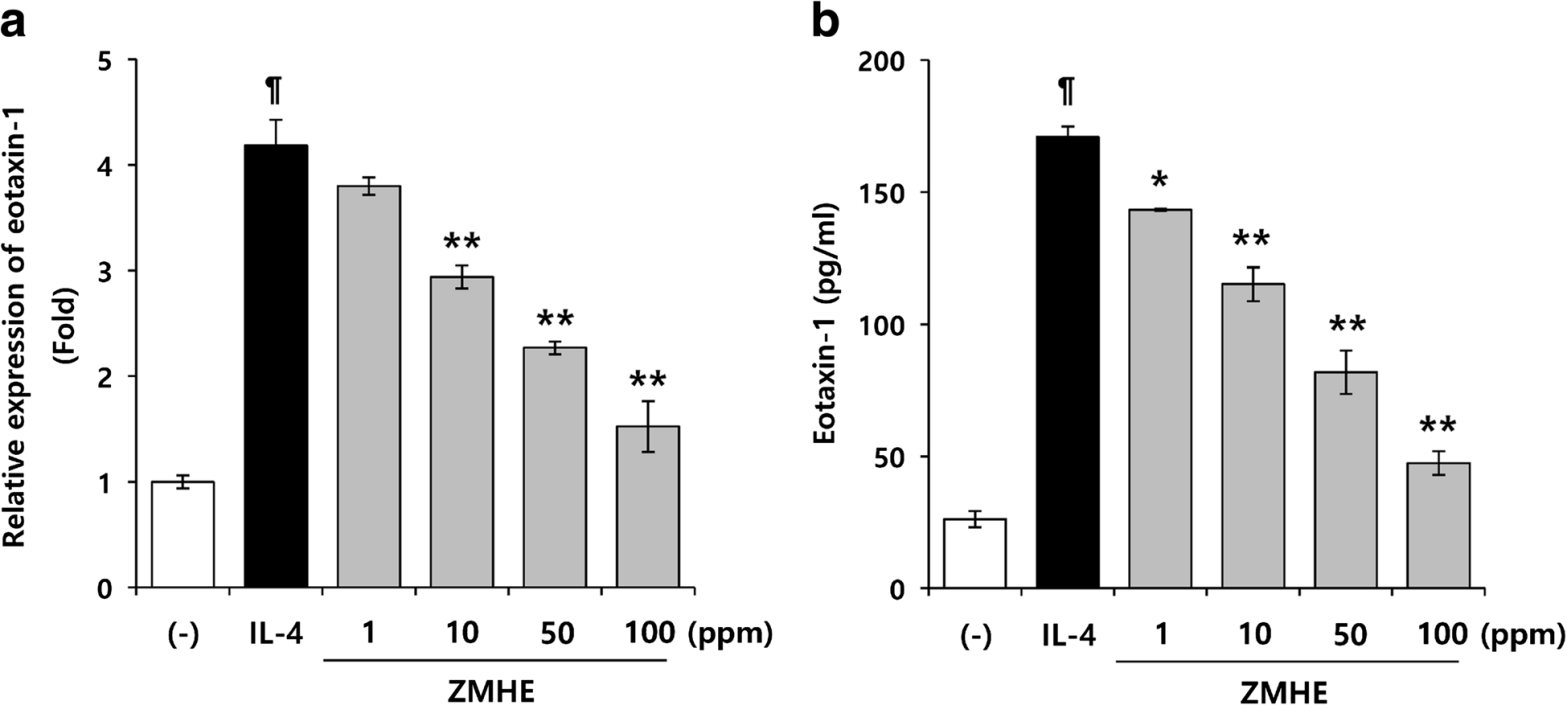
Effects of ZMHE on eotaxin-1 gene expression. **a** The eotaxin-1 luciferase reporter vector was transfected into NIH/3 T3 cells and cultured for 24 h. The cells were pretreated with ZMHE for 1 h and then stimulated with IL-4. Luciferase activity was calculated against IL-4-unstimulated control. **b** Cells were pretreated with ZMHE for 1 h and then further incubated with IL-4 (50 ng/ml) for 24 h. Eotaxin-1 release was then determined using an ELISA. The results are mean ± standard deviation (SD) (*n* = 3). *P* < 0.01 vs. IL-4-untreated control. *P < 0.05 vs. IL-4-treated control. **P < 0.01 vs. IL-4-treated control

To investigate the effect of ZMHE on eotaxin-1 expression, we first investigated the effects of ZMHE on IL-4-induced expression of eotaxin-1 gene in NIH/3 T3 cells. In this study, we used IL-4 as a stimulator which is reported to induce expression of eotaxin-1 gene in fibroblasts [14]. To measure eotaxin-1 expression, a luciferase reporter assay and an ELISA were introduced. As shown in Fig. 4a, activation of the eotaxin-1 promoter induced by IL-4 was reduced by ZMHE (1.52 ± 0.24) compared to LPS-treated group (4.18 ± 0.25). In addition, eotaxin-1 protein level was also inhibited by ZMHE (Fig. 4b). These results indicate that ZMHE downregulates expression of eotaxin-1 induced by IL-4.

### LPS-induced expression of intercellular adhesion molecule-1 is inhibited by ZMHE

**Fig. 5:**
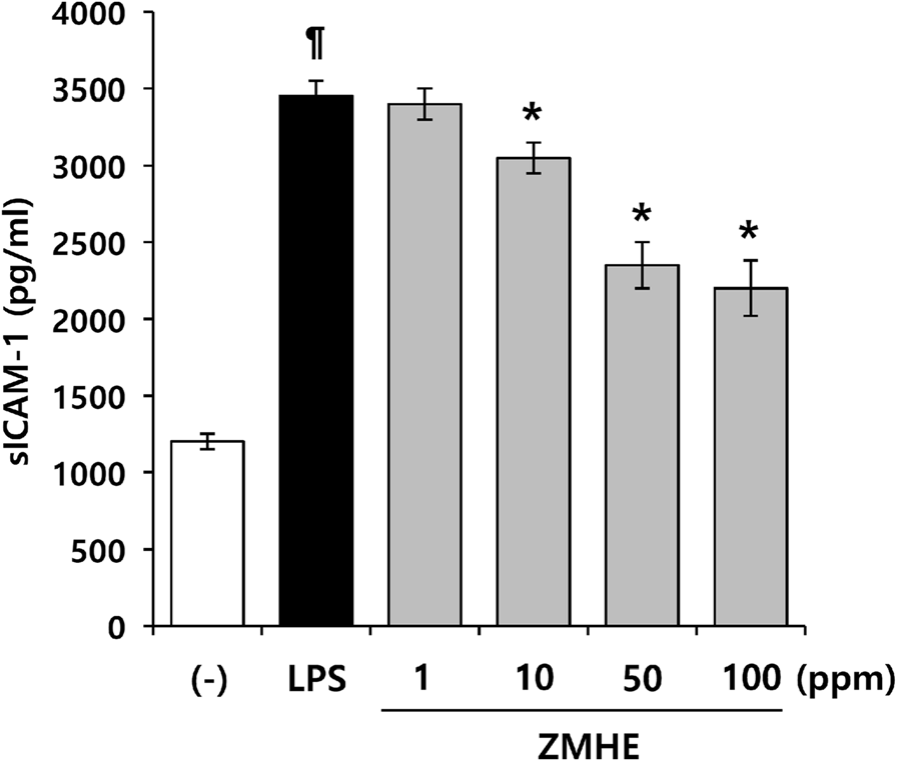
Effects of ZMHE on the expression ICAM-1. RAW264.7 cells were pretreated with ZMHE for 1 h before stimulation with LPS (200 ng/ml). After 24 h of incubation, the concentrations of sICAM-1 in the culture medium were measured by ELISA. The results are mean ± standard deviation (SD) (*n* = 3). *P* < 0.01 vs. LPS-untreated control. *P < 0.01 vs. LPS-treated control

Intercellular adhesion molecule-1 (ICAM-1) is an inducible cell surface glycoprotein belonging to immunoglobulin [15], participated in a wide range of inflammatory and immune responses [16]. Since the expression of ICAM-1 plays key role in the recruitment and extravasation of circulating leukocytes at sites of infection, it induces subsequent activation of inflammation [17, 18]. Therefore, ELISA for soluble ICAM-1 was performed to examine the involvement of ZMHE in LPS-induced expression of ICAM-1. As shown in Fig. 5, LPS-induced production of soluble ICAM-1 was significantly reduced by ZMHE in a concentration-dependent manner.

## Discussion

Although *Z. Mays* has been used as various types of human consumption, no studies have systematically examined the effects of *Z. Mays* on inflammation. In this study, the anti-inflammatory effects of *Z. Mays* extracts were demonstrated and its mechanisms of action were characterized. Specifically, among seven different aerial parts (husk, flag leaf, cob, kernel, silk, tassel, and stalk) of *Z. Mays*, ZMHE exerted the best anti-inflammatory activity by reducing the expression of pro-inflammatory mediator such as iNOS by inhibiting AP-1 and NF-kB signaling.

NO is the main macrophage-derived inflammatory mediators [19]. Aberrant control of NO production leads to an inflammatory response that induces damage to the host cells. NO is produced by a specific enzyme called nitric oxide synthase (NOS) from L-arginine. Almost every cell and many immunological parameters are modulated by NO. But, NO can be both pro- and anti-inflammatory, depending on local concentrations [20]. Abnormal overproduction of NO by iNOS under unfavorable conditions can exert harmful effects. Therefore, methods to inhibit NO production induced by inflammatory stimuli could be a useful therapeutic approach for the treatment of inflammatory diseases [21]. All together, these data indicate that NO regulates various inflammatory processes such as acute and chronic inflammation. For this reason, this study was designed to examine the effects of *Z. Mays* extracts on production of NO. In this analysis, we found that ZMHE inhibited LPS-induced production of NO in RAW264.7 cells. In addition, expression of iNOS was inhibited by ZMHE. These results indicate that ZMHE has anti-inflammatory activities by downregulating expression of iNOS gene and suggests the possibility that ZMHE can act as an anti-inflammatory agent.

In macrophages, LPS stimulation activates several intracellular signaling pathways such as the NF-kB pathway and three MAPK pathways. The MAPK family is composed of ERK, JNK, and p38 MAPKs and their activity is modulated by upstream protein kinase molecules and stress-related inducers [22]. The MAPK cascade also posttranslationally regulates activation of NF-kB and AP-1 [23, 24], which leads to the induction of many inflammatory genes. The transcription factor NF-kB has been implicated in the regulation of many immunomodulatory genes [25, 26] as well as inflammatory genes such as iNOS and COX-2. Transcription factor AP-1 also regulates expression pro-inflammatory genes and protective antioxidant genes. [27]. In addition, NF-kB and AP-1 are involved in the suppression of apoptosis and induction of cellular transformation, proliferation, invasion, metastasis, and chemo-resistance. In this study, the inhibitory mechanisms of ZMHE on the expression of iNOS gene was assessed and activation of NF-kB and AP-1 promoters induced by LPS was shown to be significantly reduced by ZMHE. These findings suggest that ZMHE downregulates expression of iNOS gene by inhibiting NF-kB and AP-1.

Taken together, the results of this study demonstrate that ZMHE has anti-inflammatory activities by downregulating the expression of iNOS gene through inhibiting NF-kB and AP-1 signaling. Additionally, these results show that ZMHE could be introduced as a potential therapeutic approach for the treatment of inflammatory diseases.

## Conclusion

ZMHE inhibited production of NO in RAW264.7 cells. In addition, ZMHE reduced expression of iNOS gene by inhibiting the NF-kB and AP-1 signaling pathway. These findings suggest that ZMHE may be used as both a soothing agent and for the treatment of inflammatory diseases.

## Abbreviations

AP-1: Activator protein-1
BSA: Bovine serum albumin
COX-2: Cyclooxygenase-2
DMSO: Dimethyl sulfoxide
ELISA: Enzyme-linked immunosorbent assay
eNOS: Endotherial nitric oxide synthase
FBS: Fetal bovine serum
GAE: Gallic acid equivalent
HRP: horseradish peroxidase
iNOS: Inducible nitric oxide synthase
ISO: International organization for standardization
KCLB: Korean cell line bank
LPS: Lipopolysaccharides
MAPKs: Mitogen-activated protein kinases
NF-kB: Nuclear factor kappa B
nNOS: Neural nitric oxide synthase
NO: Nitric oxide
QUE: Quercetin equivalent
sICAM-1: Soluble intercellular adhesion molecule-1
Z. mays: Zea mays L
ZMHE: Z. mays husk extract
